# Continuous-Flow and Scalable Synthesis of Pd@Pt_nL_ Core–Shell Nanocrystals with Enhanced Activity toward Oxygen Reduction

**DOI:** 10.1021/acs.jpcc.4c07102

**Published:** 2024-12-09

**Authors:** Helan Wang, Jianlong He, Ming Zhou, Younan Xia

**Affiliations:** † The Wallace H. Coulter Department of Biomedical Engineering, Georgia Institute of Technology and Emory University, Atlanta, Georgia 30332, United States; § School of Chemistry and Biochemistry, Georgia Institute of Technology, Atlanta, Georgia 30332, United States

## Abstract

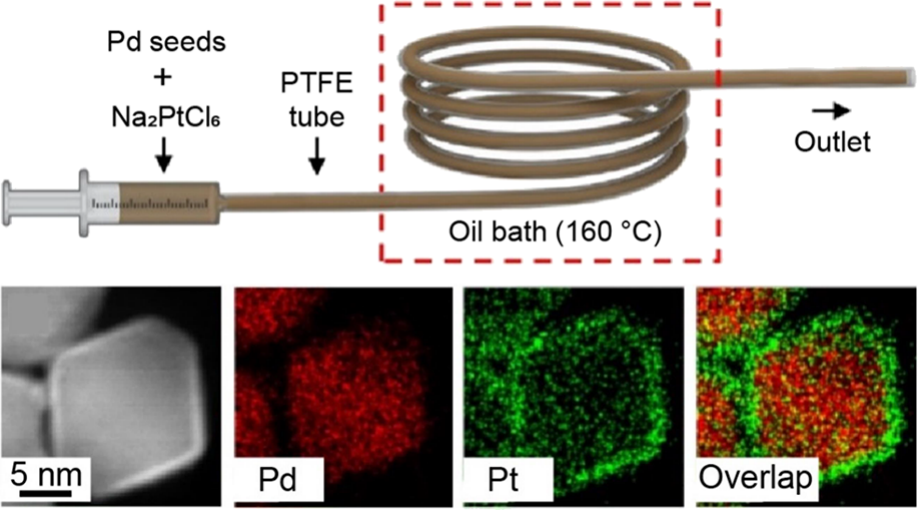

We report a scalable method based on continuous-flow reactors for conformally coating the surfaces of facet-controlled Pd nanocrystals with uniform, ultrathin shells made of Pt. The key to the success of such an approach is the identification of a proper polyol to generate the Pt atoms at a relatively slow rate to ensure adequate surface diffusion and thus the formation of uniform shells in a layer-by-layer fashion. We first demonstrate the concept using the production of Pd@Pt_nL_ (n = 2–5) core–shell icosahedral nanocrystals and then have the strategy successfully extended to the syntheses of Pd@Pt_nL_ cubic and octahedral nanocrystals. All these core–shell nanocrystals showed great enhancement in catalytic activity toward the oxygen reduction reaction. Our results suggest that seed-mediated growth can be combined with a continuous-flow reactor to achieve scalable production of bimetallic and even trimetallic nanocrystals with controlled sizes, shapes, compositions, and properties.

## Introduction

Platinum (Pt) is the most effective catalyst for accelerating the oxygen reduction reaction (ORR) key to the operation of a proton-exchange membrane fuel cell (PEMFC).^1,2^ However, owing to the scarcity, limited supply, high cost, and ever increasing demand, it remains a grand challenge to commercialize Pt-based PEMFCs at an industrial scale.^3,4^ To address this issue, there is an urgent need to increase the mass activity of a Pt-based ORR catalyst and thus reduce the necessary loading of Pt. In recent years, a number of strategies have been explored to enhance the ORR activities of Pt-based catalysts.^5−9^ Among them, depositing an ultrathin Pt layer on the surface of other metal offers an attractive strategy to increase the dispersion of Pt atoms and thus maximize the activity in terms of Pt mass.^10,11^ It has been reported that the Pt shells could be deposited on palladium (Pd) nanocrystals using an electrochemical or wet chemical approach. However, the capability of the electrochemical approach tends to be limited in terms of scalable production due to the involvement of two-dimensional electrodes.^12,13^ In a set of studies, we have demonstrated the synthesis of Pd@Pt_nL_ (n = 2–5) core–shell nanocrystals with different surface structures through a solution-phase process,^14−17^ and all of the nanocrystals exhibited remarkable enhancement in ORR activity.

Typically, the Pt shells were deposited using a syringe pump to introduce a Pt(II) or Pt(IV) precursor dropwise into a growth solution containing Pd seeds and a reducing agent. The formation of Pt shells on the Pd seeds is often troubled by homogeneous nucleation and/or island growth because the bonding energy of Pt–Pt (307 kJ mol^–1^) is much greater than that of Pd–Pt (191 kJ mol^–1^).^18^ Therefore, injection of the Pt precursor has to be kept at a slow pace in order to suppress homogeneous nucleation by maintaining the newly formed Pt atoms at a low concentration. Although uniform, well-controlled Pt shells could be formed, this method falls short of expectation because of its low production yield, long reaction time, and poor reproducibility from batch to batch. Taking the preparation of Pd@Pt_3L_ icosahedra as an example, it typically takes 6 h to obtain only 0.33 mg (in terms of Pt) solid sample from a typical batch synthesis.^16^ This small amount is far from being enough for any commercial evaluation. In addition to the slow speed, any synthesis involving dropwise injection has to be conducted in batch reactors such as flasks or vials. It is impractical to have the synthesis implemented in continuous-flow reactors.

Continuous-flow synthesis offers an attractive platform for the scalable production of noble-metal nanocrystals. Continuous-flow, defined as the flow of an uninterrupted phase running through a tube or channel, has been used in the synthesis of organic, inorganic, and biological materials.^19,20^ In terms of nanomaterials, several groups have demonstrated the synthesis of metal nanocrystals, including gold (Au), silver (Ag), copper (Cu), and cobalt (Co), via continuous flow in microfluidic devices.^21−23^ Meanwhile, the feasibility of this method has also been extended to the production of bimetallic nanocrystals. For example, Guo and co-workers synthesized platinum–bismuth (Pt–Bi) nanoparticles, and platinum–iron (Pt–Fe) and platinum–tin (Pt–Sn) nanowires in continuous-flow reactors.^24,25^ Wagner and co-workers used Au nanoparticles as seeds to grow large nanoparticles in continuous-flow reactors.^26^ However, most of these reports were based on microfluidic reactors fabricated solid substrates, which are susceptible to blockage during operation in addition to a limited throughput. To this end, we and other groups have started to explore the use of millimeter-sized reactors assembled from commercial polytetrafluoroethylene (PTFE) tubes for the continuous production of noble-metal nanocrystals with uniform sizes and controlled shapes.^27,28^ In particular, Miyakawa and co-workers synthesized Pd@Pt and Cu@Ag core–shell nanoparticles using continuous-flow reactors, but the products lacked controls over shape and facet, which are important to their catalytic activity/selectivity.^29^ On the other hand, Pd cubes, octahedra, icosahedra as well as Pd–Au, Pd–Ag nanocrystals have been prepared in the millimeter-sized droplet reactors or plug reactors.^30^ However, droplet and plug reactors always require the involvement of a carrier phase and possible separation steps, which will undoubtedly increase the difficulty and complicate the procedure of operation.

In this work, we report a simple and versatile method for the seed-mediated synthesis of core–shell nanocrystals with controlled shapes in continuous-flow reactors. The use of continuous-flow allows us to linearly scale-up the production volume without making any need to change the experimental conditions. As a proof of concept, icosahedral, cubic and octahedral Pd seeds were successfully applied to the production of Pd@Pt_nL_ core–shell nanocrystals. To address the homogeneous nucleation issue caused by the relatively high concentration of Pt precursor in the sealed PTFE tube, we employed tetraethylene glycol (TTEG) at 160 °C as a moderate reducing agent to slow down the reduction rate of the Pt precursor. Our electrochemical results demonstrated that the as-prepared Pd@Pt_nL_ core–shell nanocrystals possessed enhanced activities toward ORR relative to commercial Pt/C catalyst. The capability of using continuous-flow reactors to achieve the scalable production of core–shell nanocrystals will offer new opportunities to advance their use in a variety of applications.

## Experimental Section

### Chemicals and Materials

All chemicals were used as received from Sigma-Aldrich unless specified, including ascorbic acid (AA, 99%), sodium tetrachloropalladate(II) (Na_2_PdCl_4_, 98%), sodium hexachloroplatinate(IV) hexahydrate (Na_2_PtCl_6_·6H_2_O, 98%), potassium bromide (KBr, 99%), potassium chloride (KCl, 99%), poly(vinylpyrrolidone) (PVP, MW ≈ 55,000), hydrochloric acid (HCl, 37%), acetic acid (99.7%), ethylene glycol (EG, 99%, J.T. Baker), diethylene glycol (DEG, 99%), triethylene glycol (TEG, 99%), TTEG (99%), formaldehyde (HCHO, Fisher Scientific), ethanol (200 proof, KOPTEC), and perchloric acid (HClO_4_, 70%, PPT grade, Veritas). Deionized (DI) water with a resistivity of 18.2 MΩ·cm at room temperature was used throughout the experiments.

### Synthesis of Pd Icosahedral, Cubic, and Octahedral Seeds

The Pd icosahedra with an average edge length of 13 nm were synthesized using a previously reported protocol.^16^ Typically, 80 mg of PVP was dissolved in 2.0 mL of DEG and heated at 130 °C in an oil bath under magnetic stirring for 10 min. Meanwhile, 15.5 mg of Na_2_PdCl_4_ was dissolved in 1.0 mL of DEG and added into the above solution in one shot using a pipet. The vial was capped and maintained at 130 °C for 3 h. The solid product was collected by centrifugation, followed by washing once with acetone and twice with water, and finally dispersed in TTEG at a concentration of 0.5 mg mL^–1^, as determined using inductively coupled plasma mass spectrometry (ICP-MS), for further use.

The Pd cubes with an average edge length of 18 nm were synthesized using a previous reported protocol.^31^ Typically, 600 mg of KBr, 60 mg of AA, 105 mg of PVP were dissolved in 8 mL of water, and heated at 80 °C in an oil bath for 10 min. Subsequently, 57 mg of Na_2_PdCl_4_ was dissolved in 3 mL of water and then added into the above solution in one shot using a pipet. The vial was capped and maintained at 80 °C for 3 h. The solid product was collected by centrifugation, followed by washing three times with water, and finally dispersed in TTEG at a concentration of 2 mg mL^–1^ for further use.

The Pd cubes with an average edge length of 6 nm were obtained by reducing the amount of KBr from 600 mg to 5 mg while adding 185 mg of KCl. The solid product was dispersed in water at a concentration of 2 mg mL^–1^ for the preparation of Pd octahedra with an average length of 15 nm.^32^ In a typical synthesis, 0.1 mL of HCHO, 105 mg of PVP and 0.3 mL of the as-obtained 6 nm Pd cubes were mixed with 8 mL of water and then heated at 60 °C for 10 min. Meanwhile, 29 mg of Na_2_PdCl_4_ was dissolved in 3 mL of water and then injected into the above solution in one shot using a pipet. The synthesis was continued at 60 °C for an additional 3 h. After washing three times with water, the solid product was dispersed in TTEG at a concentration of 2 mg mL^–1^.

### Fabrication of the Continuous-Flow Reactor

**Figure 1:**
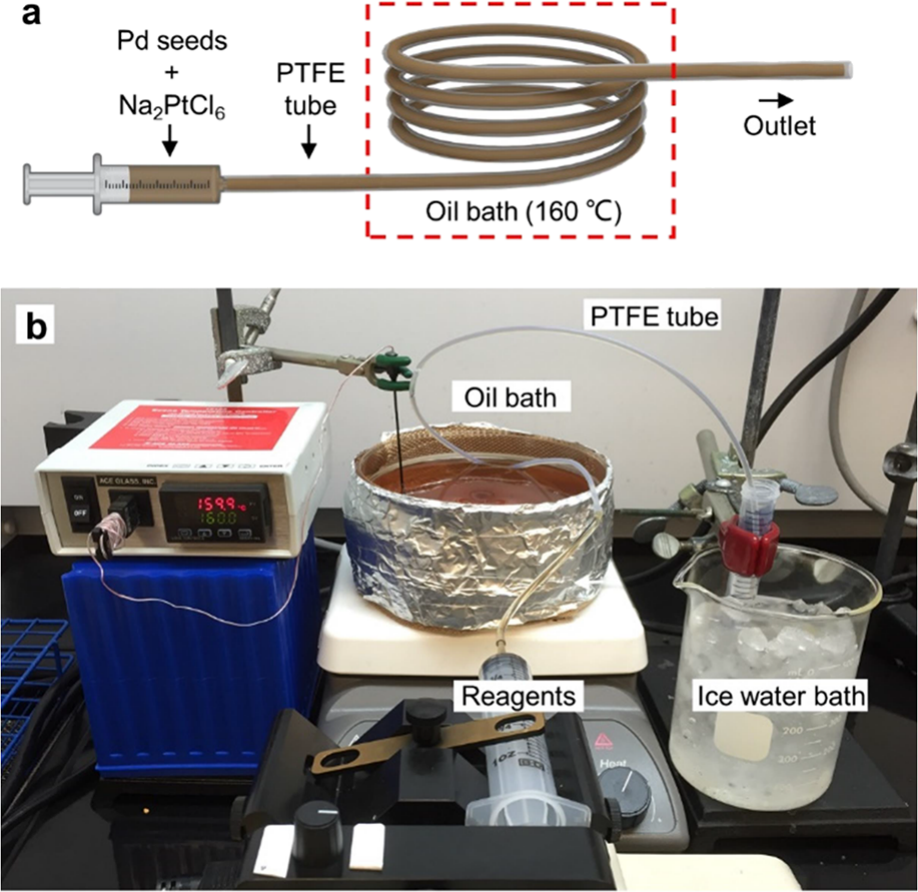
(a) Schematic illustration of the fluidic device used for the synthesis of Pd@Pt_nL_ core–shell nanocrystals in a continuous-flow manner. (b) Photography of the experimental setup.

The continuous-flow reactor was assembled from commercial components, including PTFE tube (i.d. = 1.58 mm, o.d. = 3.20 mm, length = 7 m, Sigma-Aldrich), syringe pump, and an oil bath as illustrated in Figure 1. The reaction solution was continuously introduced into the PTFE tube using a syringe pump. The reaction time could be controlled by varying the flow rate and/or the length of PTFE tube. In the current work, we turn off the pump to adjust the reaction time after a certain volume of the reaction solution had been introduced into the portion of PTFE tube inside the oil bath (i.e., the reaction zone). This simple method allowed us to use PTFE tube of 7 m in length to achieve a reaction time of 3 h in a proof- of-concept demonstration.

### Synthesis of Pd@Pt Core–Shell Nanocrystals in the Continuous-Flow Reactor

In a standard protocol for Pd@Pt icosahedra, 0.8 mg of Pd icosahedral seeds, 1.6 mg of Na_2_PtCl_6_·6H_2_O and 60 mg of PVP were mixed in 9 mL TTEG at room temperature to obtain the reaction solution. The fluidic system was operated at a flow rate of 1.0 mL min^–1^ for the reaction solution and allowed to proceed at 160 °C for 3 h, followed by collecting the mixture in a centrifuge tube immersed in an ice–water bath to terminate the reaction. The solid product was collected by centrifugation, washed once with acetone and twice with water, and then redispersed in water for further use. For the synthesis of Pd@Pt cubes or octahedra, we simply replaced the Pd icosahedral seeds with 0.9 mg of Pd cubic or 0.6 mg of Pd octahedral seeds while keeping all other conditions unchanged.

### Instrumentation

Transmission electron microscopy (TEM) images were taken using a Hitachi HT7700 microscope operated at 120 kV by dropping the nanocrystal dispersions on carbon-coated Cu grids and drying under ambient conditions. High-angle annular dark field scanning transmission electron microscopy (HAADF-STEM) and energy-dispersive X-ray spectroscopy (EDX) scanning analysis were performed using a Hitachi HD2700 microscope operated at 200 kV. The metal contents were measured using an ICP mass spectrometer (NexION 300Q, PerkinElmer).

### Electrochemical Measurements

First, the Pd@Pt nanocrystals were loaded onto a carbon support (Ketjen black EC-300J) dispersed in ethanol with a metal loading content of 20% based on the total mass of Pd and Pt (determined by ICP-MS). Typically, 0.8 mg of the nanocrystals and 3.2 mg of the carbon support were dispersed in 5 mL of ethanol under ultrasonication for 3 h. The resulting catalyst was collected by centrifugation at 12,000 rpm, redispersed in 10 mL of acetic acid, and heated at 60 °C for 12 h to clean the surface of the nanocrystals. The catalyst was then washed five times with ethanol. Once dried, 1.5 mg of the catalyst was redispersed in a mixture of 0.5 mL of isopropanol, 0.5 mL of water, and 20 μL of 5% Nafion under ultrasonication for 20 min. Then, 10 μL of the suspension was deposited on a precleaned glassy carbon rotating disk electrode (RDE, Pine Research Instrumentation) with a geometric area of 0.196 cm^2^ and dried under ambient conditions. The working electrode of a commercial Pt/C catalyst (20 wt % 3.2 nm Pt particles supported on Vulcan XC-72 carbon, Premetek Co.) was prepared using a similar procedure except for skipping the treatment in acetic acid. Electrochemical measurements were conducted using a CHI 600E potentiostat (CH Instruments). A reversible hydrogen electrode (RHE, HydroFlex, eDAQ) served as the reference. All the potentials were recorded with respect to the RHE. A Pt counter electrode based on a coiled platinum wire (Pine Research Instrumentation) was used as the counter electrode. The electrolyte was 0.1 M HClO_4_ prepared by diluting a 70% double-distilled stock solution (GFS Chemicals) with water. The cyclic voltammograms (CVs) were recorded at room temperature in a N_2_-saturated electrolyte in the potential rang of 0.08–1.1 V at a scan rate of 0.05 V s^–1^. The ORR polarization curves were measured in an O_2_-saturated electrolyte at a scan rate of 0.01 V s^–1^ and a rotation speed of 1,600 rpm. The ORR data were corrected by ohmic *i*R drop compensation and background currents.

## Results and Discussion

Figure 1 shows a schematic of the fluidic device and a photograph of the entire system. Specifically, the reaction solution containing Pd seeds and Pt(IV) precursor was driven into the PTFE tube using a syringe pump. Once the reaction solution has entered the segment immersed in an oil bath, it can be quickly heated to the desired temperature to trigger the reduction of Pt(IV) to Pt(0) atoms for conformally coating the surface of Pd seeds. The reduction and coating can be quickly terminated by collecting the reaction mixture in a centrifuge tube placed in an ice–water bath. For the current synthesis, it is unnecessary to have a mixing zone because the Pt(IV) precursor (Na_2_PtCl_6_) is not soluble in TTEG at room temperature. The reduction should not be initiated until the mixture has entered the heated zone. The reaction time can be controlled by adjusting the flow rate and the length of the tube. In a typical syntesis, the total volume of production (*V*_*total*_) can be expressed as *V*_*total*_*= V*_*rate*_ × *t*, where *V*_*rate*_ is the flow rate of the fluidic device and *t* is the duration of synthesis. We can increase the total volume of production by simply extending the duration of synthesis without any need to adjust other parameters.

**Figure 2:**
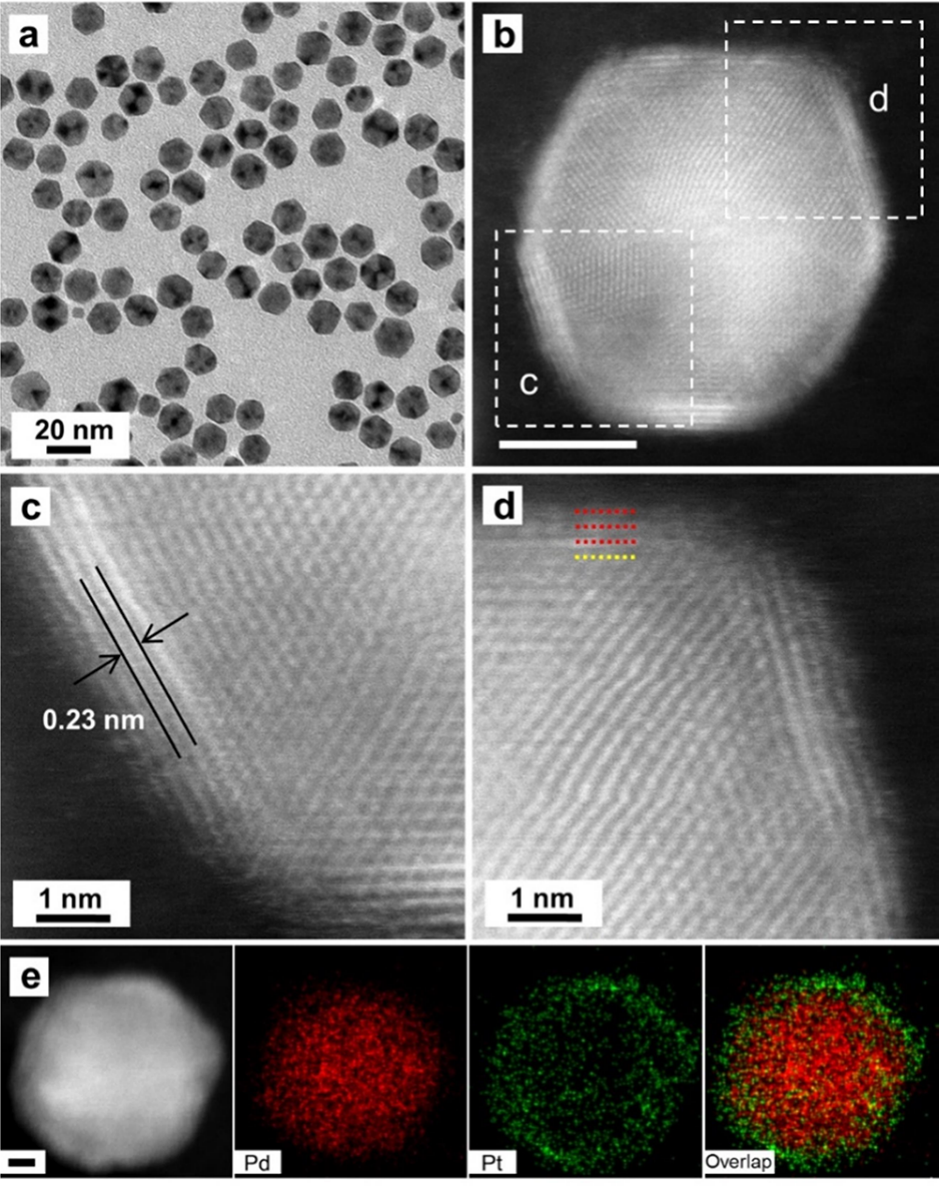
(a) TEM image of the Pd@Pt_nL_ icosahedral nanocrystals prepared using the standard protocol. (b) Atomic-resolution HAADF-STEM image taken from a single particle (scale bar: 5 nm). (c, d) Atomic-resolution HAADF-STEM image taken from the regions marked in (b). (e) EDX mapping of elemental Pd and Pt (scale bar: 2 nm).

We first applied the protocol to the coating of Pd icosahedral seeds with an average diameter of 13.0 ± 1.2 nm (Figure S1, a and b). Specifically, the synthesis was conducted at 160 °C, together with the use of Na_2_PtCl_6_ as a precursor to elemental Pt, TTEG as a solvent and a reducing agent, and PVP as a colloidal stabilizer. Figure 2a–d shows typical electron microscopy images of the Pd@Pt core–shell icosahedra. As shown by the more than 40 particles in Figure. 2a, they display the typical projected profile of an icosahedron, confirming that no island growth was involved in the Pt deposition process. As shown by the HAADF-STEM image in Figure 2b, the Pt atoms were deposited on the surface of a Pd core as a conformal, relatively uniform shell. The atomic-resolution HAADF-STEM images in Figures 2c and 2d show a lattice spacing of 0.23 nm for the shell, which matches with the {111} planes of face-centered-cubic (*fcc*) Pt. This result confirms that the icosahedral shape and {111} planes were both preserved during the Pt deposition process. On the basis of ICP-MS (Table S1), the wt % of Pt in this sample was 36.3% for a reaction time of 3 h, corresponding to an average shell thickness of about 3 atomic layers if we assume a complete, uniform Pt shell on the surface of each Pd icosahedral seed. In fact, the atomic-resolution HAADF-STEM images shown in Figures 2c, 2d, and S2 indicate that the Pt shell thickness varied in the range of 2–5 atomic layers among different facets of an individual icosahedral seed. The variation can be attributed to the lack of stirring and mixing when the Pt deposition was carried out in a sealed PTFE tube (in contrast to the traditional batch synthesis under constant magnetic stirring). As demonstrated in Figure 2e, we also analyzed the bimetallic nanocrystals by EDX mapping to confirm the formation of a Pd–Pt core–shell structure. The uniform deposition of Pt on the Pd surface can be attributed to the small (only 0.77%) lattice mismatch between Pd and Pt.^33^ By increasing the flow rate and/or the inner diameter of the PTFE tube, the production of the core–shell nanocrystals can be increased to gram-level on a daily basis. When operating multiple devices in parallel, it is even possible to go up to kg-gram production. Additionally, in our prior studies, we have demonstrated that the Pd cores could be selectively etched away to generate Pt icosahedral nanocages.^34^

**Figure 3:**
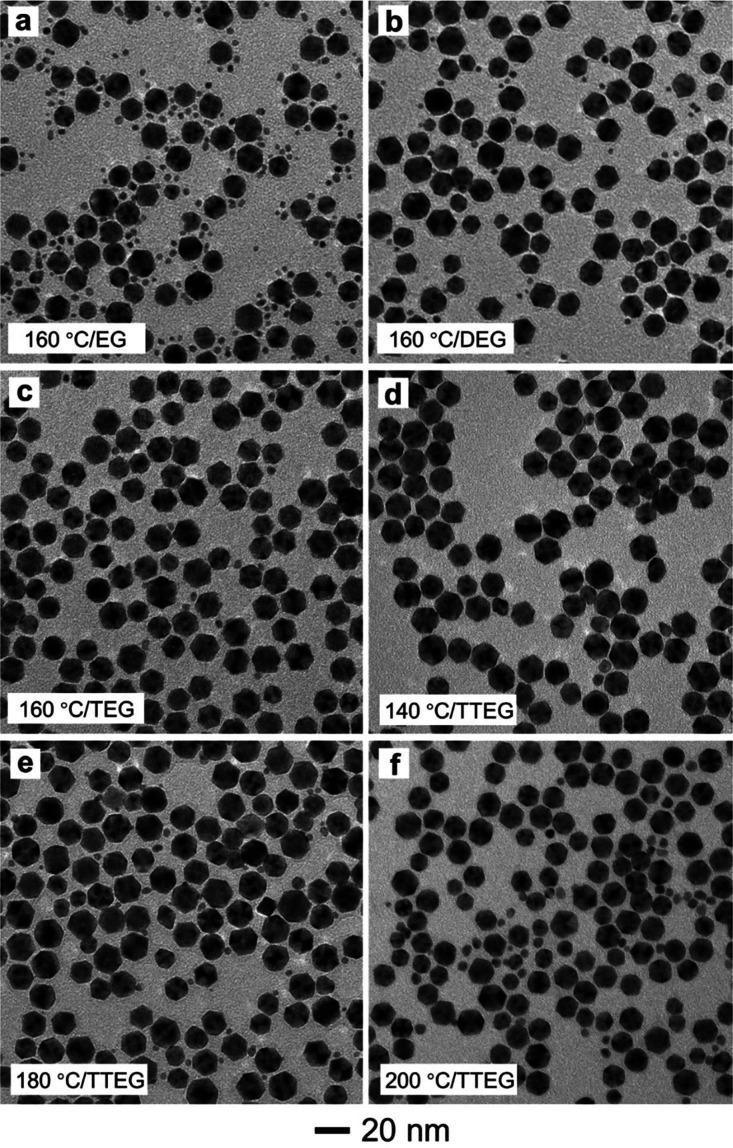
TEM images of Pd@Pt icosahedra prepared using the standard protocol, except for the variations in polyol and reaction temperature: (a) EG/160 °C, (b) DEG/160 °C, (c) TEG/160 °C; (d) TTEG/140 °C, (e) TTEG/180 °C, and (f) TTEG/200 °C, respectively.

The use of TTEG as a relatively mild reducing agent was vital to the formation of a conformal Pt shell. We investigated the conversion of the Pt(IV) precursor to Pt atoms at different time points to estimate the reaction kinetics. As shown in Table S1, the conversion of the Pt(IV) precursor reached 45.5, 69.7 and 81.6% at *t* = 20 min, 1 and 3 h, respectively, suggesting that Pt deposition occurred in the entire period of 3 h. It is the relatively slow reduction rate that suppressed homogeneous nucleation and island growth. This condition is completely different from the fast reduction process involved in the dropwise method, which was conducted in EG at a much higher temperature (200 °C vs 160 °C).^16^ As discussed in the literature, the reducing power of polyols, which originates from the hydroxyl groups, typically decreases with the elongation of the hydrocarbon chain.^35^ To understand the possible impact of reducing power on the synthesis conducted in a continuous-flow reactor, we also applied the standard procedure based on TTEG to other polyols, including EG, DEG, and TEG. Among these polyols, EG has the shortest hydrocarbon chain, and thus the strongest reducing power. The reduction rate of PtCl_6_^2–^ in EG was much faster than that in TTEG. Most of the Pt(IV) precursor was consumed in the early stage, resulting in severe homogeneous nucleation, as indicated by the small particles marked in Figure 3a. The reducing power of DEG was lower than that of EG, but still greater than that of TTEG. Therefore, the newly produced Pt atoms still tended to undergo homogeneous nucleation because of a fast reduction rate (Figure 3b). The Pt@Pd nanocrystals obtained with TEG took a concave morphology, together with a small number of Pt nanoparticles formed through homogeneous nucleation. These results clearly demonstrate that the reduction power for PtCl_6_^2–^ increases in the order of TTEG < TEG < DEG < EG.^36^ These results also suggest that the reduction power of TEG is well-suited for eliminating homogeneous nucleation and simultaneously achieving conformal deposition. When TTEG is used at an appropriate temperature, the rate for Pt atom deposition (*V*_*deposition*_) will be on par with the rate of surface diffusion (*V*_*diffusion*_), leading to the conformal deposition of Pt atoms on Pd seeds in a layer-by-layer fashion.^18^

We also studied the effect of reaction temperature on the morphology of the resultant particles. When the reaction temperature was reduced to 140 °C, the product still adopted a uniform icosahedral shape similar to that obtained at 160 °C. However, the weight percentage of Pt in the final product dropped from 36.3 to 11.5% (according to ICP-MS analysis) due to the decreased reducing power of TTEG at a lower temperature. When the reaction temperature was increased to 180 and 200 °C, the syntheses would be conducted under much faster reduction kinetics, resulting in severe homogeneous nucleation (Figure 3e and 3f). Taken together, it is clear that a proper temperature is required to ensure the adequate supply of Pt atoms without involving homogeneous nucleation and, at the same, adequate surface diffusion for the Pt adatoms on the Pd seeds. It can be concluded that continuous-flow reactors can be used to produce Pd@Pt core–shell icosahedra in a reliable and scalable fashion by leveraging the moderate reaction kinetics offered by TTEG at a temperature of 160 °C.

**Figure 4:**
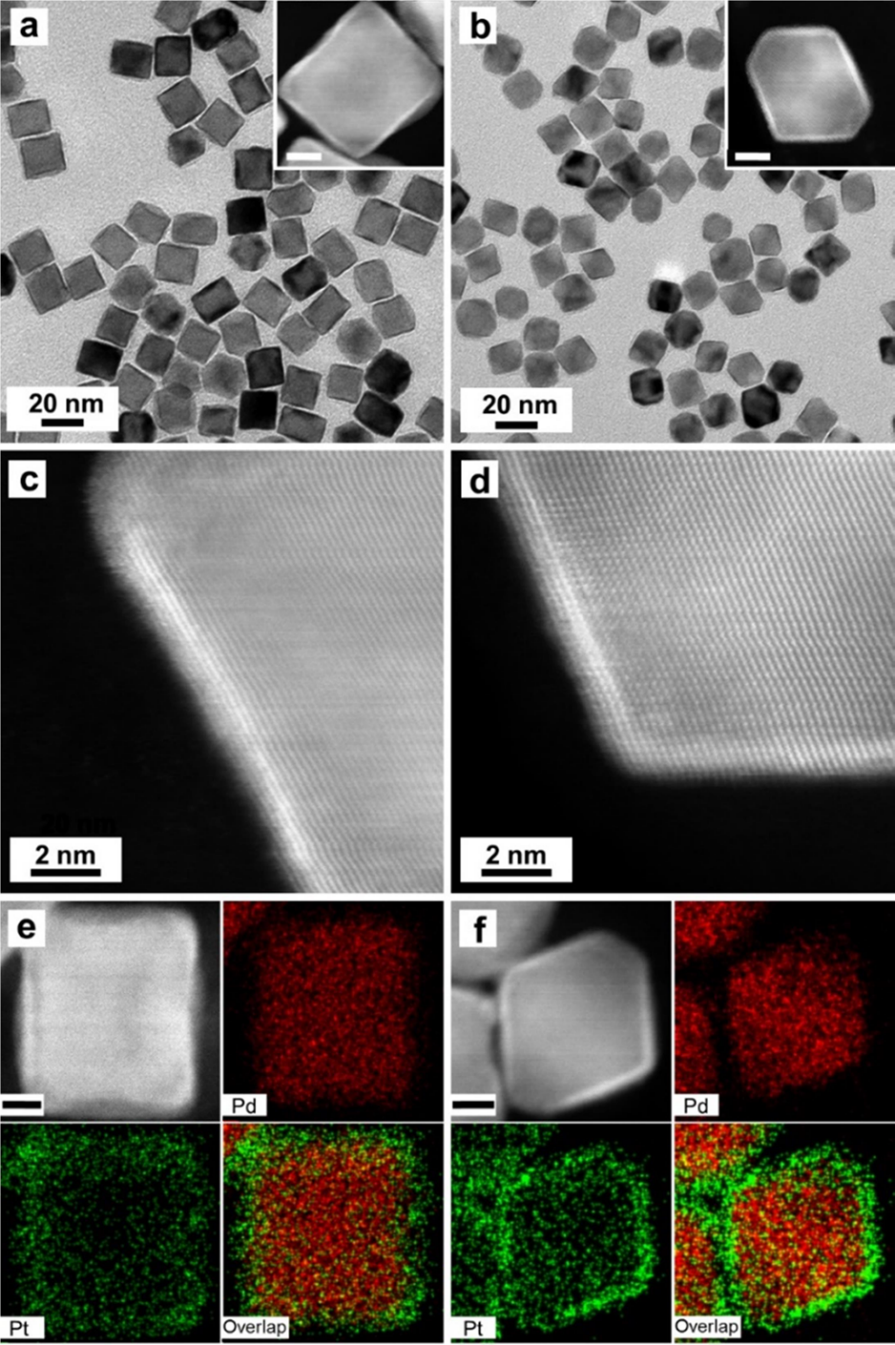
Characterizations of Pd@Pt_nL_ cubes and octahedra obtained from the 18 nm Pd cubic and 15 nm Pd octahedral seeds, respectively: (a, b) TEM images, together with atomic-resolution HAADF-STEM images in the insets (scale bars: 5 nm). (c, d) Atomic-resolution HAADF-STEM images of one particle along the edge. (e, f) HAADF-STEM image and the corresponding EDX mapping of Pd and Pt (scale bars: 4 nm).

We also conducted the synthesis in continuous-flow reactors with 18 nm Pd cubic and 15 nm Pd octahedral seeds (Figure S1, c-f) using the standard protocol developed for the Pd icosahedral seeds, except for the variation in seed number to adjust the Pt shell thickness. As shown by the HAADF-STEM image in Figure 4a and 4b, the deposited Pt can be easily identified as a bright shell around the Pd cubic or octahedral seed. Due to the use of a relatively high temperature of 160 °C, the Pd@Pt octahedra tended to show truncation at the corners. The magnified HAADF-STEM images in Figure 4c and 4d clearly show conformal Pt shells of about 4 atomic layers in thickness. The HAADF-STEM image of Pd@Pt cubes also shows a slightly concave surface, with more Pt being deposited at the corners and edges relative to the faces. This result can be attributed to the difference in activation energy barrier for a Pt adatom to diffuse across Pd(111) and Pd(100) surfaces, respectively. The barriers are 1.06 and 0.16 eV for diffusion on Pd(100) and Pd(111), differing by almost 7 folds. The diffusion of Pt adatoms on Pd(100) surface would be further retarded by the chemisorbed Br^–^ ions. These factors explain why it is so easier to obtain a conformal coating of Pt on a Pd octahedron or icosahedron via surface diffusion relative to the case of a Pd cube.^15^ The EDX mapping (Figure 4e and 4f) shows a distinct difference in color between the core and the shell, confirming the formation of a Pd@Pt core–shell structure for both the cubic and octahedral systems.

**Figure 5:**
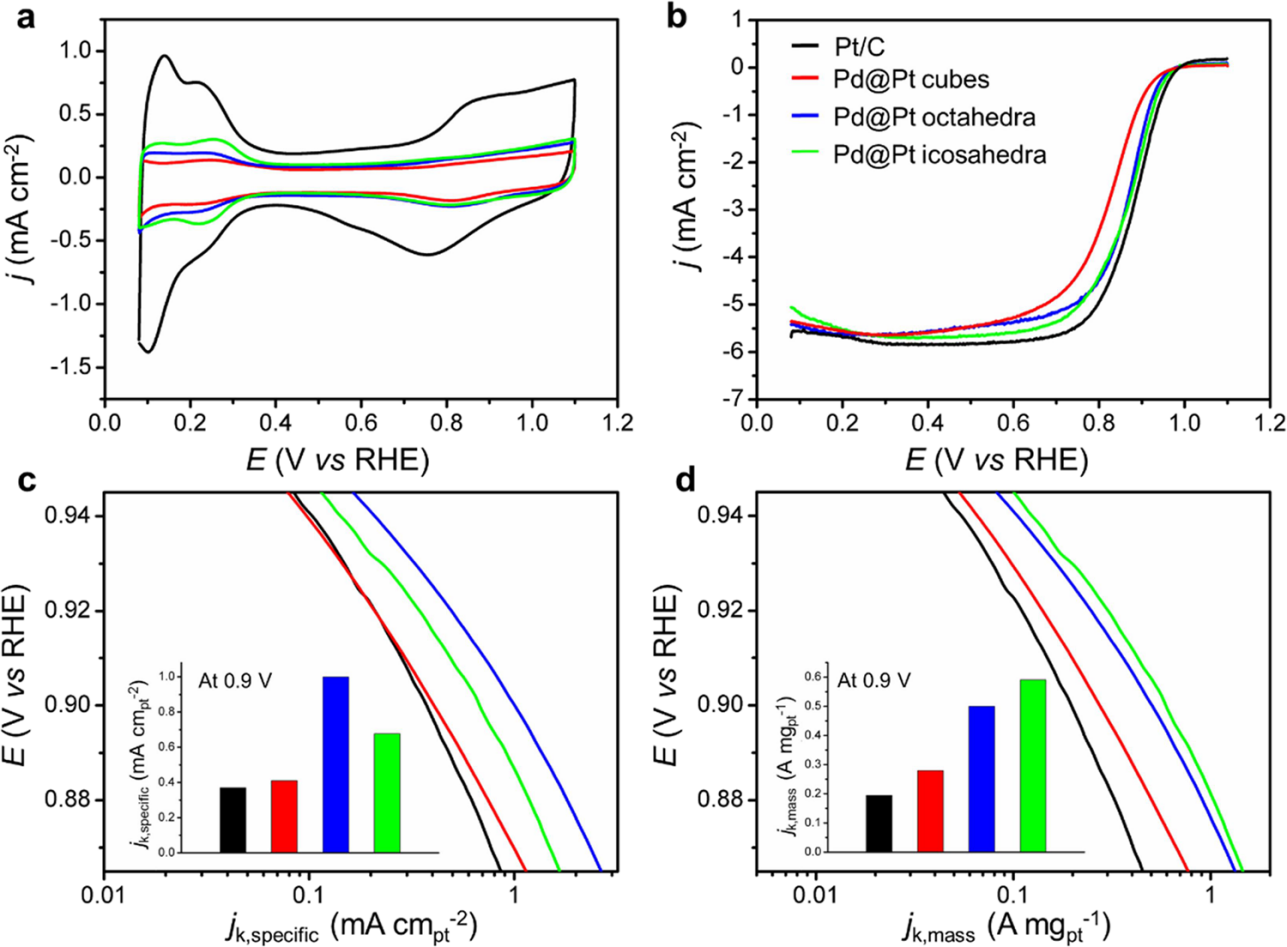
(a) Cyclic voltammograms and (b) ORR polarization curves recorded from the commercial Pt/C and Pd@Pt nanocrystal/C catalysts (including cubes, octahedra, and icosahedra). The currents were normalized to the geometric area of the RDE. (c, d) Specific and mass activities given as kinetic current densities (*j*_k_) normalized against the ECSAs and the Pt mass of the catalysts, respectively. The color scheme in (b) applies to all panels.

We further deposited the as-obtained Pd@Pt icosahedra, cubes, and octahedra on carbon and benchmarked their ORR activities against a commercial Pt/C catalyst. Figure 5a shows CVs of the four different types of catalysts. For each catalyst, we derived its electrochemical active surface area (ECSA) from the charges associated with the desorption of hydrogen between 0.08–0.45 V_RHE_. The specific ECSA of a catalyst was then obtained by normalizing against the Pt mass (Table S2). The specific ECSAs of the nanoscale cubes (67.4 m^2^ g^–1^), octahedra (50.1 m^2^ g^–1^), and icosahedra (87.4 m^2^ g^–1^) were found to be comparable to or even greater than that of the Pt/C (52.6 m^2^ g^–1^) although all these Pd@Pt nanocrystals were much larger than the Pt nanoparticles in the commercial Pt/C. This result supports our claim that the Pt atoms were better dispersed on the surface of Pd seeds as a result of the formation of ultrathin shells with a thickness of only a few atomic layers. Figure 5b shows the positive-going ORR polarization curves of the catalysts. The mass and specific activities (*j*_k, mass_ and *j*_k, specific_) were then calculated using the Koutecky–Levich equation and normalized to the corresponding ECSA and Pt mass. Relative to the commercial Pt/C, both the specific and mass activities of all the Pd@Pt/C catalysts were enhanced in the potential region of 0.86–0.94 V_RHE_ as shown in Figure 5c and 5d. Most significantly, the mass activity of the Pd@Pt_nL_ icosahedra (0.59 A mg_Pt_^–1^) showed a 3-fold enhancement relative to the commercial Pt/C (0.2 A mg_Pt_^–1^). This result is in agreement with the data reported for the Pd@Pt_nL_ icosahedra prepared in batch reactors.^16^ The ORR activity of Pd@Pt_nL_ icosahedra produced in continuous-flow reactors was slightly lower because of the variation in shell thickness among different facets of an icosahedral seed (Figure 2). Taken together, it can be concluded that the production of Pd@Pt_nL_ nanocrystals with enhanced ORR activities could be scaled up with the use of continuous-flow reactors. Although the Pd cores contained in the core–shell nanocrystals still contribute to the materials cost of a catalyst, recent studies indicate that those cores could be selectively etched away to generate Pt-based nanocages with essentially the same ORR activities.^17^

## Conclusions

In summary, we have successfully demonstrated the use of seed-mediated growth in a continuous-flow reactor for the scalable production of Pd@Pt_nL_ nanocrystals with enhanced activities toward ORR. We first established the protocol using Pd icosahedral seeds and systematically investigated the experimental conditions. The seed-mediated growth approach allows for a tight control over the Pt shell thickness for the resultant core–shell nanocrystals. The success of this synthesis relies on the identification and optimization of a set of parameters, include the type of polyol and reaction temperature. We further extended the coating procedure to the preparation of Pd@Pt_nL_ nanoscale cubes and octahedra to fully demonstrate the capability and feasibility of the continuous-flow reactor. All the Pd@Pt_nL_ core–shell nanocrystals exhibited enhanced activities toward ORR when benchmarked against the commercial Pt/C catalyst. Our results suggest that the continuous-flow reactor would become a practical platform for scaling up the production of core–shell nanocrystals through seed-mediated growth.

## Supporting Information Available

TEM images of the Pd seeds, HAADF-STEM image of two Pd@Pt icosahedra, TEM images of the Pd@PtnL/C catalysts, the Pt deposition efficiency for the Pd@Pt icosahedra, and specific ECSAs of the commercial Pt/C catalyst and the three Pd@Pt_nL_/C catalysts (PDF)
