# Supplementary material for: Continuous-Flow and Scalable Synthesis of Pd@PtnL Core–Shell Nanocrystals with Enhanced Activity toward Oxygen Reduction
Source: J Phys Chem C Nanomater Interfaces. 2024 Dec 9;128(50):21310–6. doi: 10.1021/acs.jpcc.4c07102 (PMC11664583; doi:10.1021/acs.jpcc.4c07102)
Supplement: Supplementary file 1 — jp4c07102_si_001.pdf [file jp4c07102_si_001.pdf]

Supporting Information

Continuous-Flow and Scalable Synthesis of Pd@Pt_{nL} Core–Shell Nanocrystals with Enhanced Activity toward Oxygen Reduction

Helan Wang,[†] Jianlong He,[§] Ming Zhou,[†] and Younan Xia[†,§,*]

[†]The Wallace H. Coulter Department of Biomedical Engineering, Georgia Institute of Technology and Emory University, Atlanta, Georgia 30332, United States

[§]School of Chemistry and Biochemistry, Georgia Institute of Technology, Atlanta, Georgia 30332, United States

*Corresponding author: younan.xia@bme.gatech.edu

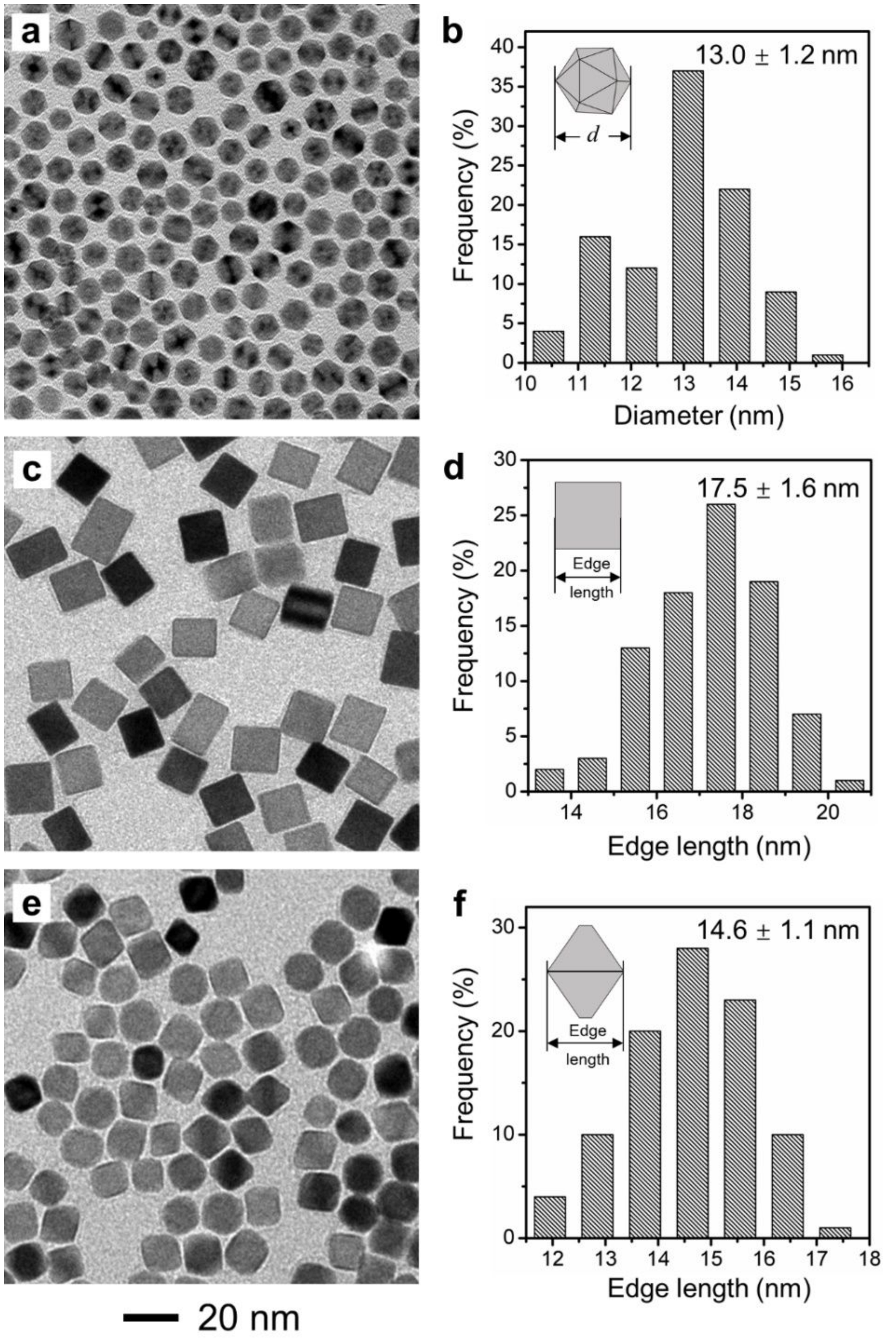

Figure S1. TEM images and size distributions of the Pd seeds with three different shapes: (a, b) icosahedra, (c, d) cubes, and (e, f) octahedra.

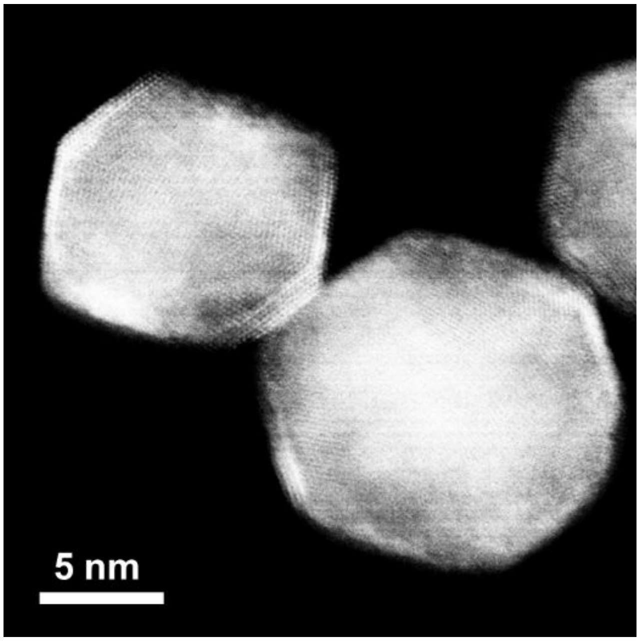

Figure S2. HAADF-STEM image of two Pd@Pt icosahedral nanocrystals, confirming the core–shell structure.

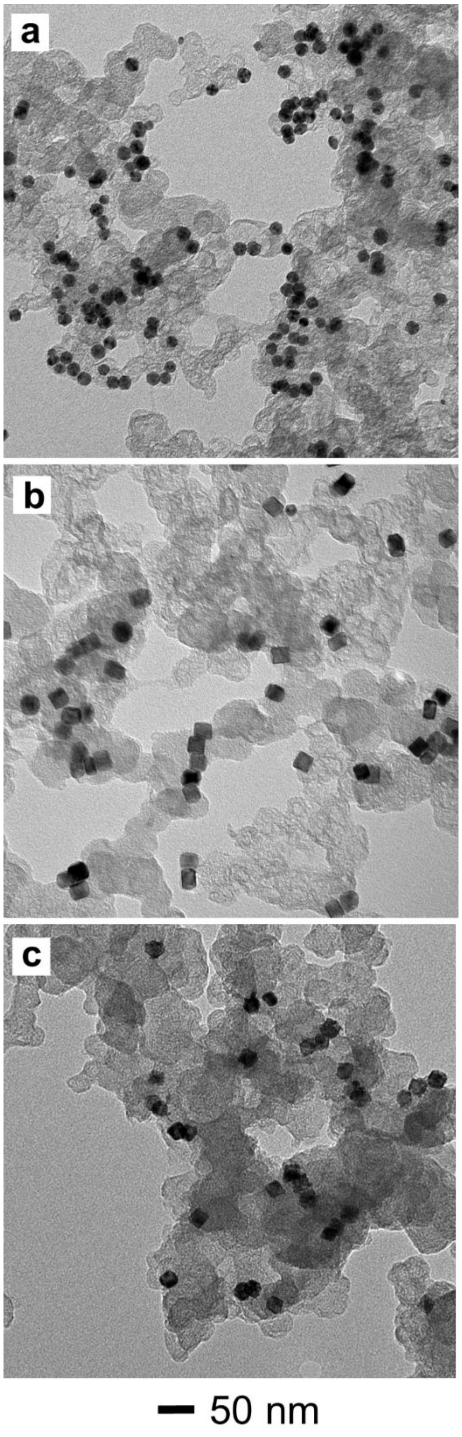

Figure S3. TEM images of the $Pd@Pt_{nL}/C$ catalysts derived from core-shell nanocrystals with different shapes: (a) icosahedra, (b) cubes, and (c) octahedra.

Table S1. The deposition efficiency (%) of Pt during the synthesis of $Pd@Pt_{nL}$ icosahedra.

Reaction time	Wt% of Pt from ICP-MS	Conversion efficiency / %
20 min	24.2	45.5
40 min	31.1	64.2
1h	32.8	69.7
2 h	35.3	77.9
3 h	36.3	81.6
4 h	36.5	82.0
5 h	36.7	82.7

Table S2. Specific ECSAs of the commercial Pt/C and three $Pd@Pt_{nL}/C$ catalysts.

	Pt/C	Pd@Pt cubes	Pd@Pt octahedra	Pd@Pt icosahedra
Specific ECSA ($m^2\ g^{-1}$)	52.6	67.4	50.1	87.4
